# Impact of universal home visits on child health in Bauchi State, Nigeria: a stepped wedge cluster randomised controlled trial

**DOI:** 10.1186/s12913-021-07000-3

**Published:** 2021-10-12

**Authors:** Khalid Omer, Altine Joga, Umar Dutse, Khalid Hasan, Amar Aziz, Umaira Ansari, Yagana Gidado, Muhd Chadi Baba, Adamu Ibrahim Gamawa, Rilwanu Mohammad, Neil Andersson, Anne Cockcroft

**Affiliations:** 1 Centro de Investigación de EnfermedadesTropicales (CIET), Universidad Autónoma de Guerrero, Acapulco, Mexico; 2 Federation of Muslim Women Association of Nigeria (FOMWAN), Bauchi, Nigeria; 3 Bauchi State Primary Health Care Development Agency, Bauchi, Nigeria; 4 CIET-PRAM, Department of Family Medicine, McGill University, Montreal, Canada

**Keywords:** Home visits, Community health workers, Child health, Childhood diarrhoea, Immunisation, Stepped wedge cluster randomised controlled trial

## Abstract

**Background:**

Nigeria is the second biggest contributor to global child mortality. Infectious diseases continue to be major killers. In Bauchi State, Nigeria, a stepped wedge cluster randomised controlled trial tested the health impacts of universal home visits to pregnant women and their spouses. We present here the findings related to early child health.

**Methods:**

The home visits took place in eight wards in Toro Local Government Authority, randomly allocated into four waves with a delay of 1 year between waves. Female and male home visitors visited all pregnant women and their spouses every 2 months during pregnancy, with a follow up visit 12–18 months after the birth. They presented and discussed evidence about household prevention and management of diarrhoea and immunisation. We compared outcomes among children 12–18 months old born to mothers visited during the first year of intervention in each wave (intervention group) with those among children 12–18 months old pre-intervention in subsequent waves (control group). Primary outcomes included prevalence and management of childhood diarrhoea and immunisation status, with intermediate outcomes of household knowledge and actions. Generalised Estimating Equations (GEE), with an exchangeable correlation matrix and ward as cluster, tested the significance of differences in outcomes.

**Results:**

The analysis included 1796 intervention and 5109 control children. In GEE models including other characteristics of the children, intervention children were less likely to have suffered diarrhoea in the last 15 days (Odds Ratio (OR) 0.40, 95% confidence interval (CI) 0.30–0.53**)** and more likely to have received increased fluids and continued feeding in their last episode of diarrhoea (OR 6.06, 95% CI 2.58–14.20). Mothers of intervention children were more likely to identify lack of hygiene as a cause of diarrhoea (OR 2.24, 95% CI 1.27–3.95) and their households had better observed hygiene (OR 3.29, 95% CI 1.45–7.45). Intervention children were only slightly more likely to be fully immunised (OR 1.67, 95% CI 0.78–3.57).

**Conclusions:**

Evidence-based home visits to both parents stimulated household actions that improved prevention and management of childhood diarrhoea. Such visits could help to improve child health even in settings with poor access to quality health services.

**Trial registration:**

ISRCTN82954580. Date: 11/08/2017. Retrospectively registered.

**Supplementary Information:**

The online version contains supplementary material available at 10.1186/s12913-021-07000-3.

## Background

Despite global progress in reducing child mortality over recent decades, an estimated 5.3 million children under age five died in 2018 [1]. Pneumonia, diarrhoea, malaria and vaccine-preventable diseases such as meningitis and measles continue to be major killers for children [2, 3]. Sub-Saharan Africa contributed roughly half the global child mortality and Nigeria was the second highest country contributor in the world [1]. The 2018 demographic and health survey in Nigeria reported an under-five mortality of 132 and infant mortality of 67 per 1000 live births [4]. The situation is worse in the Northeast of the country, including Bauchi State, where child health indicators such as prevalence of infectious diseases, immunisation coverage, hygiene and sanitation levels show the challenge of achieving the sustainable development goals [4, 5].

Well tested health interventions like immunisation can prevent many child deaths [6]. Many low- and middle-income countries have suboptimal coverage of these interventions and have a severe shortage of the workforce needed to deliver essential health services [7, 8], limiting their ability to develop and sustain adequate health systems [9]. Many childhood illnesses can be prevented or their worst consequences reduced by addressing upstream factors by changing knowledge, attitudes, behaviour and practices of care givers at home [10]. Given the inadequacy of formal health services in many low- and middle-income countries, there is recognised potential for community health workers to improve child health in these settings [11]. Community health workers can deliver a range of nutritional, maternal, neonatal and child health interventions through home visits [11].

There is a body of evidence of the beneficial effect of home visits on maternal and neonatal morbidity and mortality [12, 13]. The evidence that interventions by community health workers, including home visits, can improve child health outcomes beyond the neonatal period is less certain and reviews have highlighted the need for further randomised controlled trials [14–16]. The World Health Organization recommends increasing male involvement in reproductive health [17] and there is evidence this can improve maternal and neonatal health outcomes [18–20]. We report here the effects on child health outcomes of a trial of universal home visits to pregnant women and their spouses in Bauchi State, Nigeria.

## Methods

### Overview

A stepped-wedge cluster randomised controlled trial (RCT) tested the impact on maternal and child health outcomes of universal home visits that engaged pregnant women and their spouses [21]. The trial covered eight wards (administrative units) of Toro local government area (LGA) of Bauchi state, Nigeria. An epidemiologist not involved in the fieldwork randomly allocated wards into annual waves of intervention. An analysis after the first year of implementation of the intervention in wave 1 wards documented a significant positive impact on maternal health outcomes [22] and further analysis found a significant impact on male knowledge and attitudes (Anne Cockcroft, personal communication, 10 December 2020). This article focuses on the impact of the universal home visits on prevention and management of childhood diarrhoea and child immunisation, and on intermediate outcomes of parental knowledge and home care practices.

### Setting

Bauchi State in north-eastern Nigeria has a population of around 5 million, based on projections from the 2006 national census. The main religion is Islam, family sizes are large, and polygamy is common. Some 63% of women and 44% of men in Bauchi have no education, compared with 35 and 22% nationally [4]. Toro is the largest LGA in Bauchi, with a projected population of 487,100 living in 18 wards. More than 95% of the population is Muslim and predominantly Hausa (80%) or Fulani (12%) ethnicity. The 2018 Demographic and Health Survey [4] reported only 20% of children aged 12–23 months had received all basic vaccinations in Bauchi State, and the two-week prevalence of diarrhoea among children under 5 years was 34%. A 2013 survey found 14% of children aged 12–23 months in Toro LGA had received all basic vaccinations and 31% of children less than 5 years of age had diarrhoea during 2 weeks prior to the survey [23].

### Trial design and participants

**Fig. 1:**
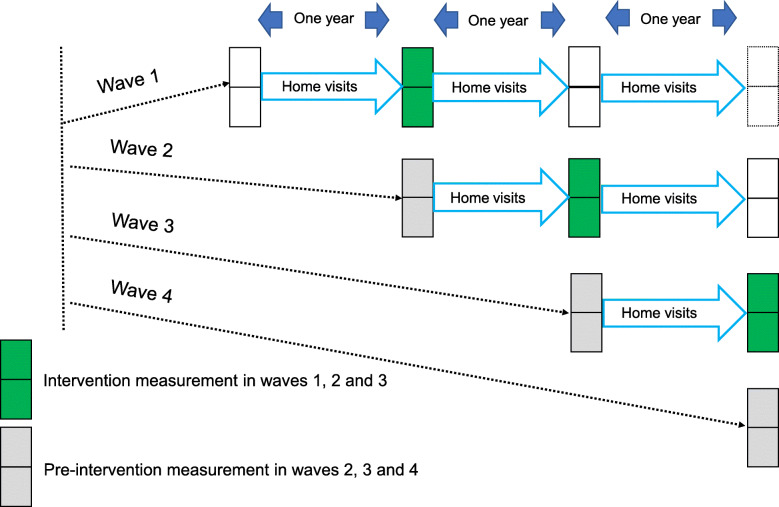
Stepped wedge design of the trial. Each ward is represented by a square box with two wards in each wave. The intervention measurement was made on children aged 12-18 months born to pregnant women visited during the first one year of home visits implementation in waves 1, 2 and 3, in a follow up visit 12-18 months after the birth. The pre-intervention (control) measurement was made on children aged 12-18 months identified in a pre-intervention visit to all households in waves 2, 3 and 4

The overall trial methods appear in detail in the published protocol [21]. The stepped wedge cluster randomised design involved a sequential crossover of pairs of clusters (wards) from control to intervention, so that that each pair of wards after the initial one began in as a control and later received the intervention, with the order of inclusion randomly determined. Fig. 1 shows the trial design. Each sequential wave receiving the intervention comprised two wards. We selected the eight wards included in the trial from the pool of 18 wards in Toro LGA, excluding wards where the security situation was precarious. Each ward included urban, rural and rural remote communities. The home visits intervention began immediately upon allocation in the initial two wards (wave 1) and began in subsequent waves of two wards after 1 year of implementation in the previous wave.

We measured child health outcomes among children born to pregnant women who were visited in households during the first year of intervention in each wave, in a follow up visit when these children had reached 12–18 months of age. We compared them with child health outcomes among children aged 12–18 months old identified in pre-intervention visits to households in the subsequent waves (serving as controls). The last two wards (wave 4) did not go on to receive the intervention. We did not collect data about child health outcomes for children aged 12–18 months old in wave 1 wards before the home visits began in these wards. Thus, wave 1 wards only contributed information on child health outcomes to the intervention group and wave 4 wards only contributed information on child health outcomes to the control group. Wave 2 and wave 3 wards contributed child outcome information to both the intervention and the control group. Because the two visits for data collection were at least a year apart, the children included in the control and intervention groups in wave 2 and wave 3 wards were different.

Fieldworkers administered a questionnaire to collect socio-demographic characteristics of the mother and other household members at the pre-intervention visits in wards in waves 2, 3 and 4 and in the first intervention visit in wave 1 wards.

### Intervention

The trial protocol includes a detailed description of the home visits intervention [21]. The research team recruited most of the home visitors to work within their own home communities, guided by community, ward and LGA leaders. The table in Additional file 1 shows characteristics of the female and male home visitors in the different waves. Most of the home visitors were married and aged 25 years or above. More male than female home visitors had post-secondary education. Characteristics of the home visitors were similar across waves, except that those in wave2 tended to be older and fewer of them had post-secondary education.

We conducted a separate training for home visitors for each wave, just before the start of the intervention in that wave. To ensure the consistency and quality of the training across all waves, the same team of trainers conducted all the trainings using a standardised curriculum. Each training over 11 days included classroom sessions and field practice. The training stressed the importance of conducting all visits and interactions with privacy and covered practical ways of ensuring privacy in the household setting in Bauchi. We trained more potential home visitors than we needed and evaluated them to select only those who had attained the required understanding and level of skills.

Each team of one female and one male home visitor had a catchment area of about 300 households. This was sometimes a whole community in rural areas, or part of a bigger urban site. The wards had between 8 and 27 catchment areas, covering all households in the ward. There were 51 catchment areas in total across all the wards. The female home visitors visited every household in their catchment areas every 2 months during daylight hours. During the first visit they registered the household and collected information about the demographic and socio-economic status of the household and listed all women of childbearing age (14–49 years). During subsequent two-monthly visits the female home visitors checked for any new pregnancies and registered and followed all pregnant women during the pregnancy. The male home visitors visited the spouses of the pregnant women, also two-monthly, at a convenient time, usually in the evening or on weekends.

On each visit the home visitors interacted with the registered pregnant women and their spouses using a surveillance questionnaire and a structured discussion guide. The discussion guide for both women and men included information about early childhood health and care, specifically covering prevention and management of diarrhoea and routine immunisation. Using the guide, the home visitors gave the pregnant women and their spouses’ information about prevention of diarrhoea, including improving household hygiene and management of drinking water containers, and about home management of childhood diarrhoea, including giving extra fluids, continuing feeding, and avoiding medicines to stop the diarrhoea. They also gave information about the benefits of childhood immunisation. They asked what actions women, their spouses and other family members could take to prevent childhood diarrhoea, to manage diarrhoea when it happened, and to ensure immunisation of children. They concluded by asking about progress with actions planned by the women and their spouses during the previous visit. In one of the two wards in each wave, the home visitors supported their discussions in the household with short video-docudramas. We will examine the potential added value of these videos in a separate analysis among the intervention group only.

The repeated evidence-based interactions with women and their spouses aimed to support them to make informed decisions and take actions which would lead to improved outcomes for the child. For example, they would gain knowledge about the connection between hygiene and the risk of childhood diarrhoea, discuss within the household how to improve hygiene, and then take actions to improve hygiene, leading to a reduction in childhood diarrhoea. The conceptual framework for the home visits intervention was based on the CASCADA results chain [24], a version of the theory of planned behaviour [25]. It proposes increasing Conscious knowledge, changing Attitudes, deviating from unhelpful Subjective norms, intention to Change, perceived Agency to change, Discussion about change, and finally Acting to implement change.

### Data collection and outcomes

The home visitors collected information from mothers (or other main carers) of children aged 12–18 months old. For children in the intervention group (born during the first 1 year of the home visits in each wave), they collected the information in a follow up visit when the child was 12–18 months old. For children in the control group, they collected this information about children aged 12–18 months old identified in the pre-intervention visits. They collected information on child health status and caring practices during the child’s first year of life as well as knowledge, attitudes and practices of the woman and her spouse about child health and care. They administered an electronic questionnaire, recording responses on GPS-enabled android handsets, and uploading records to a central server using a cellular connection. We used open-source Open Data Kit (ODK) software for this electronic data collection [26]. The background meta-data automatically stamped each record with the GPS location, the visit date, and the duration of the interaction. This enabled quality control, to check if the home visitors conducted interviews appropriately in the intended households.

#### Socio-economic characteristics

The fieldworkers collected information about education of the father, education of the mother and food security of the mother on the first home visit to households in wards in wave 1 and in the pre-intervention visit to households in wards in waves 2, 3, and 4.

#### Diarrhoea prevention and management

The primary outcomes were prevalence of diarrhoea in the last 15 days and prevalence of severe diarrhoea (with blood in the stool) within the last 15 days. Intermediate outcomes included the mother’s knowledge about poor hygiene as a cause of diarrhoea, reported treatment of drinking water, and the state of household hygiene and the water container, as observed by the home visitors.

Mothers reported on their management of the child’s last episode of diarrhoea, including whether the child received increased fluids and continued feeding during the episode and whether the child was given any anti-diarrhoeal medicine. Intermediate outcomes included the mother’s knowledge about management of diarrhoea: increased fluids and continued feeding and avoidance of anti-diarrhoeal medicine.

#### Immunisation

The home visitors asked if each child had received routine vaccinations, including the number of doses for multi-dose vaccinations. They did not request to see the child’s vaccination card. The indicator of immunisation status was whether the child was fully immunised: a fully immunised child had received the full course of BCG, Pentavalent DTP-hepB-Hib (Diphtheria, Tetanus, Pertussis, Hepatitis-B, Haemophilus Influenza), Polio, Yellow fever and Measles vaccines. Intermediate outcomes were the mother’s perception about the value of immunisation, discussions about immunisations in the family, and the mother’s involvement in the decision about immunisation of the child.

### Sample size

We calculated the trial sample size using the clinical trials simulator of Taylor and Bosch [27] and based on maternal health outcomes [22]. For child health outcomes, there were 1796 children in the intervention group and 5109 in the pre-intervention (control) group (see Table 2). Analysis of the stepped wedge trial used six clusters in the intervention arm and six in the control arm. With a prevalence of diarrhoea in the last 2 weeks of 34% in control clusters, the trial could detect a reduction of 12% with 80% power at the 5% significance level.

### Randomisation and masking

We divided the eight participating wards into two geographically separated sets of four wards each. An epidemiologist not involved in the field implementation of the trial (NA) created four waves of two wards, with one ward from each of the two sets of wards in each wave. He randomly assigned the four waves to the sequence for implementation of the home visits, using a computer-generated random sequence.

It was not possible to blind home visitors or pregnant women and their spouses to the intervention status once the implementation began. We standardised the procedures of administering the questionnaire about child health outcomes and the questionnaire used in both the intervention and pre-intervention (control) visits was the same. Fieldworkers had no reason to conduct the process differently in intervention and pre-intervention visits. Each householder gave informed consent to receive visits and to respond to questionnaires, without being aware if they were allocated to a treatment or control group.

### Consent

We followed the principles embodied in the Declaration of Helsinki to conduct the trial. We shared the study protocol and design in non-technical terms with each participating community, ward and LGA leadership and sought their approval before starting the trial. The home visitors sought and recorded oral informed consent from each household, woman and spouse to be visited and to respond to questionnaires.

### Statistical methods

Statistical analysis used CIETmap open-source software, [28] which interfaces with the R programming language. Ward was the unit of randomisation, of intervention and analysis. We used the Mantel-Haenszel procedure [29] to examine differences in characteristics between the intervention and control groups potentially related to the outcomes.

To assess the impact of home visits on pre-defined primary and intermediate child health outcomes we undertook logistic regression using Generalised Estimating Equations (GEE) [30], using an exchangeable correlation matrix and ward as cluster. For each outcome, we first ran the model including only the intervention and cluster. We then repeated the analysis, beginning with a saturated model including the six variables potentially related to the outcome and measured in intervention and control groups: urban/rural location, sex of the child, education of the mother, age of the mother, education of the father, food security of the mother. We used the same set of six variables in saturated models for all outcomes. The analysis stepped down variable by variable to a model where all variables were significantly associated with the outcome. We report the robust odds ratios (OR) and 95% confidence intervals (CIs) from the final GEE models. The intervention measurement included only children born within the first 1 year of intervention in all waves, so exposure time was equal for all the waves in the analysis.

To examine the effect of not having pre-intervention outcomes data for wave 1 wards, and not having post-intervention outcomes data for wave 4 wards, a sensitivity analysis included only wards in wave 2 and wave 3. As for the main analysis, the initial GEE model for each outcome included ward as cluster and other characteristics of the children potentially related to the outcomes.

## Results

### Participants flow

**Fig. 2:**
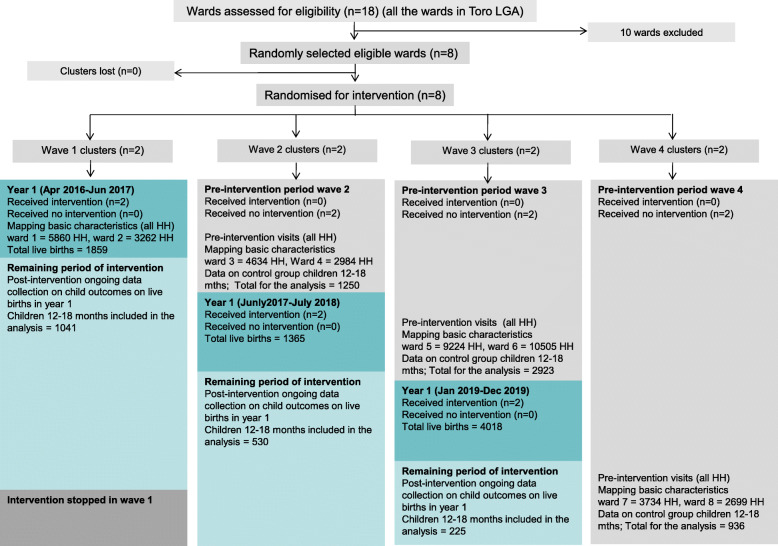
Trial flow chart by allocated sequence and period. Dark blue blocks are the first year of the periods of intervention. Light blue blocks are the periods of intervention after the first year (not included in the analysis). During this period fieldworkers collected data on children born during the first period when they reached the age of 12-18 months. Grey blocks are the pre-intervention periods in waves 2, 3 and 4. HH= household

Figure 2 shows the flow of clusters and participants into the intervention and pre-intervention (control) groups for each wave during the trial.

### Recruitment and number analysed

Table 1 shows the implementation schedule of the intervention (home visits) in waves 1–3, and the cut-off dates for the first year of intervention in each wave. The intervention began at about yearly intervals across the waves, and in waves 2 and 3 began with a baseline survey.

**Table 1:** Intervention implementation schedule

	Wave 1	Wave 2	Wave 3
Start date	March–April 2016	July–August 2017	January 2019
Cut-off for first year of intervention	June 2017	July 2018	December 2019

We analysed data on child health outcomes among 6905 children: 1796 in the intervention group, and 5109 in the control group. Table 2 shows the trial flow of participants. There were 7242 live births in total during the first year of intervention in waves 1–3. The large number of live births from wave 3 reflects the large population size of the two wards in wave 3. But few of these children had reached 12 months of age before the end of the project. The number of children in the pre-intervention (control) group from wave 3 also reflects the large population size of this wave. A few children died before they reached 12 months old, and others were lost to follow-up.

**Table 2:** Trial flow of participants

	Wave 1	Wave 2	Wave 3	Wave 4	Total
*Intervention group*					
Live births^a^	1859	1365	4018	N/A	7242
Deaths^b^	2	17	6	N/A	25
< 12 months^c^	0	0	3765	N/A	3765
Lost to follow-up^d^	816	818	22	N/A	1656
Net for analysis^e^	1041	530	225	N/A	1796
*Control group (pre-intervention)*					
	N/A	1250	2923	936	5109

^a^ The number of children born during the first year of the intervention in each wave. As expected from the larger number of households in wave 3, there were more live births during the first year of intervention in wave 3 than in the equivalent period in wave 1 and wave 2

^b^ The number of children that died soon after birth or before they reached 12 months old

^c^The number of children born during the first one year of intervention in the wave who had not reached 12 months old by the time of the end of the overall project fieldwork, so did not have outcomes measured. This only occurred for children born during the intervention period in wave 3

^d^The number of children eligible for measurements who did not have the follow up visit to make the measurements. The reasons included: some households moved away from the ward; in some areas there was a high turnover of female visitors and by the time new workers could complete the follow-up the children were over 18 months old; and in some areas security concerns limited the ability to make the follow-up visits

^e^ The net number of children included in the intervention group for the analysis is the number of live births minus the deaths, minus the < 12 months, and minus the lost to follow up

### Characteristics of children in intervention and control groups

Table 3 shows characteristics of children and their households in the intervention and control groups potentially related to the child outcomes. Compared with the control group, the intervention group had a higher proportion of children with adolescent mothers (age 14–19 years), a lower proportion of children from urban settings, and a lower proportion of children with mothers and fathers having some formal education.

**Table 3:** Characteristics of children included in the analysis in intervention and control groups

Characteristics	Intervention Percent (n/N)	Control Percent (n/N)	OR (95% CI)
Total number of children	1796	5109	
Mean age of the children (months)	13.8 (SD = 1.7)	14.2 (SD = 2.0)	
Female children	47.7 (857/1796)	47.0 (2401/5109)	0.97 (0.87–1.08)
From urban communities (vs rural & rural remote)	25.7 (461/1796)	51.0 (2604/5109)	**0.33 (0.29–0.37)**
From female headed households	0.3 (6/1738)	0.5 (26/5038)	0.67 (0.11–1.50)
With adolescent mothers (14–19 years)	14.5 (260/1796)	5.9 (303/5109)	**2.68 (2.24–3.21)**
With mothers having some formal education	39.2 (699/1784)	51.0 (2604/5101)	**0.62 (0.55–0.69)**
With fathers having some formal education	46.2 (820/1774)	56.1 (2830/5042)	**0.67 (0.60–0.75)**
With mothers having enough food in the last week	96.6 (1622/1679)	97.2 (4938/5078)	0.81 (0.59–1.15)

*OR* Odds ratio, *95% CI* 95% Confidence Interval

Bold font indicates a difference significant at the 5% level

The characteristics of the children lost to follow up were very similar to those of the children included in the analysis (Additional file 2). The only significant difference was that children included in the analysis were more likely to be from urban communities than those who were lost to follow up.

### Outcomes and estimation of impact

#### Prevention of diarrhoea

Table 4 shows the results of GEE analysis for outcomes of diarrhoea prevention. The associations between the intervention and outcomes were similar when GEE modelling included only the intervention with ward as cluster, and when the GEE initial models included other variables potentially related to the outcomes (shown in Table 3). Variables remaining in the final GEE models are shown in Additional file 3. Mothers of children in the intervention group were significantly less likely to report the child had diarrhoea within the last 15 days compared with mothers of children in the control group (OR 0.40, 95% CI 0.30–0.53). The proportion of children with recent diarrhoea in the intervention group was about half that in the control group (17% versus 34%). Mothers of children in the intervention group were also less likely to report the child had suffered severe diarrhoea in the last 15 days (OR 0.22, 95% CI 0.11–0.42). Intermediate outcomes were also better in the intervention group. Mothers of children in the intervention group were more than twice as likely to identify lack of hygiene as a cause of childhood diarrhoea (OR 2.24, 95% CI 1.27–3.95). The households of children in the intervention group were more likely to have good environmental hygiene (OR 3.29, 95% CI 1.45–7.45), to have a safe drinking water container (OR 4.09, 95% CI 2.32–7.07), and to treat their drinking water in some way (OR 3.13, 95% CI 1.76–5.59).

**Table 4:** GEE modelling for intervention effect on prevalence of diarrhoea and on intermediate outcomes in children aged 12–18 months in intervention and control groups

Outcomes	Proportion (n/N)		Robust OR (95% CI)	
	Intervention	Control	Modelled with intervention alone^a^	Modelled with other characteristics^b^
*Primary outcomes*				
Diarrhoea in the last 15 days	0.173 (270/1562)	0.341 (1517/4444)	**0.38 (0.28–0.52)**	**0.40 (0.30–0.53)**
Bloody diarrhoea in the last 15 days	0.015 (23/1559)	0.063 (280/4411)	**0.21 (0.11–0.41)**	**0.22 (0.11–0.42)**
*Intermediate outcomes*				
Mother mentions lack of hygiene as a cause of diarrhoea	0.583 (1031/1767)	0.412 (2102/5105)	**2.25 (1.29–3.93)**	**2.24 (1.27–3.95)**
Household has better hygiene (no garbage, sewage or excreta observed)	0.665 (900/1353)	0.413 (2052/4965)	**3.72 (2.10–6.59)**	**3.29 (1.45–7.45)**
Household with clean, covered and raised drinking water container observed	0.550 (744/1353)	0.244 (1210/4965)	**4.09 (2.36–7.09)**	**4.09 (2.32–7.07)**
Household treats drinking water	0.364 (490/1347)	0.167 (828/4948)	**3.13 (1.76–5.59)**	**3.13 (1.76–5.59)**

*OR* Odds Ratio, *95% CI* 95% confidence interval; Bold font indicates a difference significant at the 5% level

The lower denominators for the primary outcomes of diarrhoea and bloody diarrhoea in the last 15 days in both intervention and control groups are because some mothers could not specify when the child last had an episode of diarrhoea, and these children were excluded from the analysis

^a^ The GEE model for each outcome included only the intervention with ward as cluster

^b^ The GEE initial saturated model for each outcome included the intervention with other variables potentially related to the outcome shown in Table 3, as well as ward as cluster. The variables remaining in the final model for each outcome are shown in Additional file 3. For all outcomes, the intervention variable remained in the final model. For some outcomes no other variable remained in the final model

#### Management of diarrhoea

Table 5 shows the results of GEE analysis for outcomes of management of childhood diarrhoea. The associations between the intervention and outcomes were similar when GEE modelling included only the intervention with ward as cluster, and when the GEE initial models included other variables potentially related to the outcomes (shown in Table 3). Variables remaining in the final GEE models are shown in Additional file 3. Children in the intervention group were more likely to have received increased fluids and continued feeding during their last episode of diarrhoea, compared with children in the control group (OR 6.06, 95% CI 2.58–14.20). About 44% of children in the intervention group received increased fluids and continued feeding, and only about 10% of those in the control group. Children in the intervention group were also much more likely to have escaped receiving an anti-diarrhoeal medicine in their last episode of diarrhoea (OR 10.17, 95% CI 3.87–26.76). About one half the intervention children with diarrhoea (51%, 138/270) and almost all control children with diarrhoea received an anti-diarrhoeal medicine (93%, 1399/1511). The mothers of children in the intervention group had more informed beliefs about management of childhood diarrhoea. Mothers of children in the intervention group were more likely to know the correct management of diarrhoea (increased fluids and continued feeding) (OR 4.35, 95% CI 2.53–7.50) and to say they would not give a child anti-diarrhoeal medicine (OR 75.11, 95% CI 6.24–534.30).

**Table 5:** GEE modelling for intervention effect on management of diarrhoea and on intermediate outcomes in children aged 12–18 months in intervention and control groups

Outcomes	Proportion(n/N)		Robust OR (95% CI)	
	Intervention	Control	Modelled with intervention alone^a^	Modelled with other characteristics^b^
*Primary outcomes*^c^				
Given more fluids and continued feeding during last episode of diarrhoea	0.437 (118/270)	0.098 (147/1502)	**6.06 (2.58–14.20)**	**6.06 (2.58–14.20)**
Not given any medicine to stop diarrhoea during last episode of diarrhoea	0.489 (132/270)	0.074 (112/1511)	**9.21 (3.71–22.89)**	**10.17 (3.87–26.76)**
*Intermediate outcomes*				
Mother thinks child with diarrhoea should be given more fluid and continued feeding	0.396 (700/1767)	0.114 (584/5105)	**4.35 (2.53–7.50)**	**4.35 (2.53–7.50)**
Mother would not give a child medicines to stop diarrhoea	0.366 (647/1767)	0.031 (157/5105)	**12.53 (5.82–26.96)**	**75.11 (6.24–903.18)**

*OR* Odds Ratio, *95% CI* 95% confidence interval; Bold font indicates a difference significant at the 5% level

^a^ The GEE model for each outcome included only the intervention with ward as cluster

^b^ The GEE initial saturated model for each outcome included the intervention with other variables potentially related to the outcome shown in Table 3, as well as ward as cluster. The variables remaining in the final model for each outcome are shown in Additional file 3. For all outcomes, the intervention variable remained in the final model. For some outcomes no other variable remained in the final model

^c^ Among children with diarrhoea in the last 15 days

#### Child immunisation

Table 6 shows the results of GEE analysis for the outcomes of child immunisation. Children in the intervention group were slightly more likely to be fully immunised than those in the control group but the difference was not significant at the 5% level, whether the intervention effect was modelled alone or with other variables potentially related to the outcomes (shown in Table 3). Mothers of children in the intervention group were more likely to report being involved in decisions about childhood immunisation than mothers of children in the control group, but the difference was not significant at the 5% level (OR 1.77, 95% CI 0.89–3.54).

**Table 6:** GEE modelling for intervention effect on immunisation status and on intermediate outcomes in children aged 12–18 months in intervention and control groups

Outcomes	Proportion(n/N)		Robust OR (95% CI)	
	Intervention	Control	Modelled with intervention alone^a^	Modelled with other characteristics^b^
*Primary outcome*				
Fully immunised	0.498 (894/1795)	0.468 (2389/5103)	1.80 (0.82–3.95)	1.67^c^ (0.78–3.57)
*Intermediate outcomes*				
Mother thinks it is worthwhile to immunise children	0.971 (1712/1763)	0.970 (4913/5065)	1.25 (0.52–2.98)	1.52^c^ (0.59–3.91)
Mother discusses immunisation with spouse and family	0.930 (1644/1767)	0.922 (4705/5105)	1.73 (0.69–4.32)	2.57^c^ (0.79–8.36)
Mother involved in decision about immunising the child	0.262 (469/1789)	0.143 (730/5094)	1.77 (0.89–3.54)	1.77^c^ (0.89–3.54)

*OR* Odds Ratio, *95% CI* 95% confidence interval

^a^ The GEE model for each outcome included only the intervention with ward as cluster

^b^ The GEE initial saturated model for each outcome included the intervention with other variables potentially related to the outcome shown in Table 3, as well as ward as cluster. The variables remaining in the final model for each outcome are shown in Additional file 3

^c^ The OR and 95% CI are those from the initial GEE model including the intervention with other variables; the intervention variable was not in the final model

#### Sensitivity analysis

Additional file 4 shows the results of the GEE analysis for all outcomes, excluding the wards in wave 1 and wave 4. The findings were similar to those shown in Tables 4, 5 and 6, which included all the wards.

#### Harms

We did not receive any reports of harm arising from the home visits during the trial. In focus group discussions after the intervention period, participants from visited households confirmed that the home visits did not cause any problems for them. Some said that sharing the same information with both women and their spouses avoided potential conflicts. (Loubna Belaid, personal communication, 20 December 2020).

## Discussion

The young children of mothers who received the home visits intervention were significantly less likely to suffer diarrhoea than those of mothers without the intervention. Mothers of children in the intervention group had a better understanding of the role of poor hygiene as a cause of diarrhoea and their observed household hygiene was better. Intervention children were also significantly more likely to have correct home management when they did suffer diarrhoea, and their mothers had better knowledge of home management of diarrhoea.

Other studies in Africa have reported an impact of home visits on childhood diarrhoea. A trial in Malawi found that volunteer counselling delivered through home visits led to a significant reduction in reported infant cough, fever, and diarrhoea [31]. A recent trial in Ghana reported that home visits from community health volunteers reduced child diarrhoea and fever in communities with high coverage and regular household contacts of effective duration [32]. A trial in India of home visits by community-based workers failed to demonstrate an effect on child cough, fever, or diarrhoea prevalence [33]. The primary focus of the intervention in India was on nutrition and child growth; education on diarrhoea prevention care and hygiene was part of counselling to prevent malnutrition.

Children in the intervention group in our study were only slightly more likely to be fully immunised, and the association with the intervention was not statistically significant, possibly because the home visits programme did not address availability of or access to vaccines. A trial of home visits in an urban setting in Ghana reported a 20% increase in immunisation coverage in the intervention group [34]. Their home visits included direct referral to immunisation clinics and follow-up of those who did not attend the clinics after the initial visits. In Karachi, a short educational intervention about immunisation delivered in home visits in addition to routine advice from community health workers increased immunisation uptake by 39% [35]. A Cochrane review of interventions to improve coverage of childhood immunisations in low- and middle-income countries concluded that education in communities or home visits could probably increase coverage, but the evidence was of low certainty [36]. A systematic review of demand-side interventions to improve immunisation uptake noted that the impact of such interventions is limited by supply-side factors such as difficult access to facilities and non-availability of vaccines in local clinics [37]. A trial of evidence-based community discussions in Pakistan led to an increase in immunisation coverage; some communities took action to support individual households to access immunisation services [38]. Our home visits trial did not include any intervention to improve access to facilities or the quality of service available there. Adding improvements in the supply-side to the home visits might lead to a significant increase in immunisation coverage.

In our study the home visitors used local evidence [23] about risk factors for child health actionable at household level to spark discussions with expectant mothers and their spouses. This created an opportunity for spouses to communicate with each other on the evidence, leading to improved knowledge and actions by the households themselves to address these upstream factors. The improvements in intermediate outcomes of knowledge and attitudes about diarrhoea prevention and management that we demonstrated to be associated with the home visits support the idea that household actions, such as improving hygiene arrangements, led to improved child health outcomes. This is in line with our conceptual framework for the home visits, based on the CASCADA results chain [24]. We have used a similar approach of socialising evidence for participatory action in successful trials of community mobilisation in other settings [38–40].

Community Health Extension Workers in the Nigerian primary health care system are supposed to spend 60–80% of their time on community-based activities, including home visiting [41]. In practice, due to shortages of nurses, midwives, and physicians, they spend 60 to 80% of their time in health facilities [42]. The Nigerian federal ministry of health has developed a Community Health Influencers and Promoters programme to create a new cadre of community volunteers to implement community-based activities, including home visits [43]. The programme has not yet been implemented. The improvements in child health associated with the home visits reported here and in maternal health reported elsewhere [22] support the potential benefit of such a national initiative. Our experience with implementing a successful programme of universal home visits in one LGA could inform state and national rollout.

### Strengths and limitations

The design as a cluster randomised controlled trial, with an analysis adjusting for clustering by ward and for the potential confounding effects of other characteristics of the children, increases our confidence that measured differences were due to the home visits intervention. Engaging the spouses of the pregnant women probably helped the implementation of household changes that improved child health outcomes, and universal coverage of the home visits ensured inclusion of the most marginalized households.

Mothers self-reported their knowledge and behaviours for prevention and management of childhood diarrhoea, with the possibility of reporting bias among visited mothers. Self-reports were supplemented by fieldworkers’ observation of hygiene conditions and the state of visible drinking water containers, and these indicators were also better in the visited households. Immunisation status of children relied on maternal reporting; evidence from low- and middle-income countries suggests that such reporting is reliable [44]. The home visitors collected the information on child health outcomes, and it is possible that mothers or caregivers in visited wards gave socially desirable responses or simply repeated what they had discussed with the home visitors. But there is no reason to believe mothers in visited wards would under-report the prevalence of diarrhoea in their children and its severity.

The limited number of clusters in the intervention and control groups raises the possibility of an imbalance of unmeasured cluster-level covariates between the groups. Such imbalance is less likely because all the clusters were in one local government area, which is quite homogeneous in terms of religion, ethnicity and socio-economic status. In the stepped-wedge design, wards in waves 2 and 3 contributed data in both pre-intervention and intervention status. Our sensitivity analysis, excluding wave 1 and wave 4 data, gave similar findings to the main analysis including data from all wards (see Additional file 4). We did not include calendar time as a covariate in the analysis. Analysts recommend including calendar time in the analysis of stepped wedge trials because in most cases the unexposed observations will, on average, be from an earlier calendar time than exposed observations, so improvements over time due to external factors could mean calendar time is a confounder [45]. In our analysis, 87.5% (1571/1796) of the exposed observations were from earlier waves (1 and 2) and 75.5% (3859/5109) of the unexposed observations were from later waves (3 and 4) (see Table 2), so any general secular trend in the outcomes would have reduced the measured impact of the intervention.

Many children were lost to follow-up after the intervention in waves 1 and 2. We list reasons for the difficulty in following-up children born to mothers during the intervention year in the footnote to Table 3. None of these would systematically exclude children with worse health outcomes. The only important difference between children lost to follow up and those included in the analysis was that those included in the analysis were more likely to be from urban communities (see Additional file 2). Across both intervention and control groups, children from rural communities were less likely to have had diarrhoea within the last 2 weeks than those from urban communities (1095/4269 vs 913/2837, OR 0.73, 95% CI 0.65–0.81), so the relative lack of rural children in the intervention group because of loss to follow up might have diluted the measured intervention effect.

Other donor-supported initiatives took place in Bauchi State, including in Toro LGA, during the period of our home visits intervention, and some of them targeted child health outcomes measured in our study. Additional file 5 gives a brief description of these initiatives. Most of them focused on improving services in health facilities, and those active during the period of the home visits were implemented in all wards of the LGA so would not have differentially improved outcomes in the intervention group in our study.

## Conclusion

This stepped wedge randomised controlled trial in West Africa provides evidence in support of a positive impact of home visits on important child health outcomes. The likely mechanism is that the evidence-based home visits and discussions with both parents changed knowledge and stimulated household actions to improve child health outcomes. Home visits programmes engaging mothers and their spouses may help to improve child health even in settings with poor coverage and quality of facilities-based health services.

## Supplementary Information

**Additional file 1: Table A1.** Characteristics of female and male home visitors by wave. Shows the characteristics of female and male home visitors employed in the home visit project

**Additional file 2: Table A2.** Characteristics of children included in the analysis and lost to follow up. Shows the characteristics of children included in the analysis and those lost to follow-up

**Additional file 3.** Final GEE models for outcomes shown in Tables 4, 5 and 6 of the main text. The file includes tables showing all the final GEE models for outcomes shown in Tables 4, 5 and 6 of the main text. Variables remaining in final models of GEE that began with saturated models including the characteristics of children in intervention and control groups potentially related to the outcomes

**Additional file 4.** Results of sensitivity analysis, excluding data from wards in wave 1 and wave 4. The file contains tables showing results from sensitivity analysis on the same outcome indictors included in the main manuscript but excluding data from wards in wave 1 and wave 4

**Additional file 5.** Other initiatives in Toro LGA potentially related to the measured child health outcomes. This file contains a description and relevant web links to view details about other initiatives in Toro LGA during the period of the home visits

## Data Availability

The datasets used and/or analysed during the current study are available from the corresponding author on reasonable request.

## Abbreviations

GEE: Generalised Estimating Equations
RCT: Randomised controlled trial
GPS: Global Positioning System
LGA: Local Government Authority
ODK: Open Data Kit
WHO: World Health Organisation
