# Supplementary material for: Impact of universal home visits on child health in Bauchi State, Nigeria: a stepped wedge cluster randomised controlled trial
Source: BMC Health Serv Res. 2021 Oct 12;21:1085. doi: 10.1186/s12913-021-07000-3 (PMC8513291; doi:10.1186/s12913-021-07000-3)
Supplement: Supplementary file 1 — Additional file 1: Table A1. Characteristics of female and male home visitors by wave. Shows the characteristics of female and male home visitors employed in the home visit project [file 12913_2021_7000_MOESM1_ESM.pdf]

Table A1: Characteristics of female and male home visitors by wave

Characteristic	Female visitors [n/N (%)]			Male visitors [n/N (%)]		
	Wave 1	Wave 2	Wave 3	Wave 1	Wave 2	Wave 3
Aged 25 years or less	15/42 (35.7)	5/29 (17.2)	26/60 (43.3)	6/33 (33.7)	1/18 (5.6)	10/47 (21.3)
Currently or ever married	29/42 (69.0)	25/29 (86.2)	42/60 (70.0)	17/33 (51.5)	14/18 (77.8)	20/47 (42.6)
Post-secondary education	14/42 (33.3)	2/29 (6.9)	28/60 (46.7)	28/33 (84.8)	9/18 (50.0)	36/47 (76.6)
Some health-related training	7/42 (16.7)	0 (0)	13/60 (21.7)	19/33 (57.6)	12/18 (66.7)	12/47 (25.5)
Some other employment	11/42 (26.2)	8/29 (27.6)	4/60 (6.7)	20/33 (60.6)	12/18 (66.7)	11/47 (23.4)
Higher income occupation of household head	19/42 (45.2)	10/29 (34.5)	26/60 (43.3)	8/33 (24.2)	2/18 (11.1)	33/46 (71.7)
Some social or political status in community	11/42 (26.2)	9/29 (31.0)	10/60 (16.7)	17/33 (51.5)	17/18 (94.4)	37/47 (78.7)
Reside in their catchment area	10/42 (23.8)	18/29 (62.1)	27/60 (45.0)	12/33 (36.4)	15/18 (83.3)	32/47 (68.1)
Recommended by community representative	11/42 (26.2)	10/29 (34.5)	24/60 (40.0)	8/33 (24.2)	7/18 (38.9)	31/47 (66.0)
Recommended by government officer or political leader	31/42 (73.8)	19/29 (65.5)	36/60 (60.0)	25/33 (75.8)	11/18 (61.1)	16/47 (34.0)
Worked as home visitor for >1 year	24/42 (57.1)	19/29 (65.5)	55/60 (91.7)	29/32 (90.6)	16/17 (94.1)	36/44 (81.8)
