# Supplementary material for: Impact of universal home visits on child health in Bauchi State, Nigeria: a stepped wedge cluster randomised controlled trial
Source: BMC Health Serv Res. 2021 Oct 12;21:1085. doi: 10.1186/s12913-021-07000-3 (PMC8513291; doi:10.1186/s12913-021-07000-3)
Supplement: Supplementary file 2 — Additional file 2: Table A2. Characteristics of children included in the analysis and lost to follow up. Shows the characteristics of children included in the analysis and those lost to follow-up [file 12913_2021_7000_MOESM2_ESM.pdf]

Table A2: Characteristics of children included in the analysis and lost to follow-up

Characteristics	Percent(n/N)		OR (95%CI)
	Included in analysis	Lost to follow-up	
Total number of children	1796	1656	
Female children	47.7 (857/1796)	50.3 (825/1639)	0.90 (0.79 – 1.03)
From urban communities (vs rural & rural remote)[1]	25.7 (461/1796)	10.8 (179/1656)	**2.85 (2.36 – 3.48)**
From female headed households	0.3 (6/1738)	0.5 (8/1596)	0.69 (0.14 – 2.40)
With adolescent mothers (14-19 years)	14.5 (260/1796)	14.4 (239/1656)	1.00 (0.83 – 1.22)
With mothers having some formal education	39.2 (699/1784)	41.0 (679/1656)	0.93 (0.81 – 1.07)
With fathers having some formal education	46.2 (820/1774)	47.8 (748/1566)	0.94 (0.82 – 1.08)
With mothers having enough food in the last week	96.6 (1622/1679)	95.8 (1585/1655)	1.26 (0.87 – 1.84)

Bold font indicates a difference significant at the 5% level.
95%CI = 95% confidence Interval; OR=Odds ratio.
[1] Children lost to follow-up were less likely to be from urban communities because there were relatively more children lost to follow up in wave 2 (see Table 2 in main text) and wave 2 has relatively few urban communities
