# Supplementary material for: Impact of universal home visits on child health in Bauchi State, Nigeria: a stepped wedge cluster randomised controlled trial
Source: BMC Health Serv Res. 2021 Oct 12;21:1085. doi: 10.1186/s12913-021-07000-3 (PMC8513291; doi:10.1186/s12913-021-07000-3)
Supplement: Supplementary file 3 — Additional file 3. Final GEE models for outcomes shown in Tables 4, 5 and 6 of the main text. The file includes tables showing all the final GEE models for outcomes shown in Tables 4, 5 and 6 of the main text. Variables remaining in final models of GEE that began with saturated models including the characteristics of children in intervention and control groups potentially related to the outcomes [file 12913_2021_7000_MOESM3_ESM.pdf]

Additional file 3: final GEE models for outcomes shown in Tables 4, 5 and 6 of the main text

All models include Ward as cluster

Variables included in initial models:
Male sex of child
From urban community
From female headed household
Mother had enough food in last week
Mother adolescent (aged 14-19)
Mother with some formal education
Father with some formal education

Final models relevant to findings shown in Table 4 in main text

Table S3.1 Final GEE model for outcome of:Diarrhoea within last 15 days

Variable	OR	95% CI
Home visits intervention	0.39892	0.29964 – 0.53108
Mother had enough food in last week	0.56327	0.38360 – 0.82709

Table S3.2 Final GEE model for outcome of:Bloody diarrhoea within last 15 days

Variable	OR	95% CI
Home visits intervention	0.21546	0.11108 – 0.41709
Mother had enough food in last week	0.36604	0.22869 – 0.58590

Table S3.3 Final GEE model for outcome of:Mother mentions lack of hygiene as a cause of diarrhoea

Variable	OR	95% CI
Home visits intervention	2.24454	1.27387 – 3.95484
Mother with some formal education	1.41680	1.17633 – 1.70643

Table S3.4 Final GEE model for outcome of: Household has better hygiene (observed)

Variable	OR	95% CI
Home visits intervention	3.28675	1.44920 – 7.45428
From urban community	0.58036	0.44057 – 0.76451
Mother with some formal education	1.64757	1.47399 – 1.84159

Table S3.5 Final GEE model for outcome of: Household has clean, covered and raised drinking water container (observed)

Variable	OR	95% CI
Home visits intervention	4.08823	2.32291 - 7.19513
Mother had enough food in last week	1.47523	1.11018 - 1.96031

Table S3.6 Final GEE model for outcome of: Household treats drinking water

Variable	OR	95% CI
Home visits intervention	3.13338	1.75593 - 5.59137

No other variable remained in the final model

Final models relevant to findings shown in Table 5 in main text

Table S3.7 Final GEE model for outcome of: Given more fluids and continued feeding during last episode of diarrhoea

Variable	OR	95% CI
Home visits intervention	6.05783	2.58374 - 14.20315

No other variable remained in the final model

Table S3.8 Final GEE model for outcome of: Not given any medicine to stop diarrhoea during last episode of diarrhoea

Variable	OR	95% CI
Home visits intervention	10.17174	3.86576 - 26.76428
Mother with some formal education	1.69634	1.28981 - 2.23099

Table S3.9 Final GEE model for outcome of: Mother thinks child with diarrhoea should be given more fluid and continued feeding

Variable	OR	95% CI
Home visits intervention	4.35359	2.52469 – 7.50733

No other variable remained in the final model

Table S3.10 Final GEE model for outcome of: Mother would not give a child medicines to stop diarrhoea

Variable	OR	95% CI
Home visits intervention	75.11348	6.23997 – 903.17597
From urban community	4.36972	1.55914 – 12.24684

Models relevant to findings shown in Table 6 in main text

Note: These are the initial models. The intervention variable was not significantly associated with the outcome in any of the initial or final models.

Table S3.11 Initial GEE model for outcome of: Child fully immunised

Variable	OR	95% CI
Home visits intervention	1.66729	0.77814 – 3.57244
Male sex of child	1.01217	0.91877 – 1.11508
From urban community	1.06588	0.52223 – 2.17546
From female headed household	0.99243	0.55318 – 1.78046
Mother had enough food in the last week	1.53849	0.99160 – 2.38700
Mother adolescent (aged 14-19)	0.88303	0.68818 – 1.13305
Mother with some formal education	1.35053	1.10863 – 1.64522
Father with some formal education	1.28377	1.00088 – 1.64661

Table S3.12 Initial GEE model for outcome of: Mother thinks it is worthwhile to immunise children

Variable	OR	95% CI
Home visits intervention	1.68220	0.66461 – 4.25778
Male sex of child	0.83879	0.71369 – 0.98581
From urban community	0.78836	0.40218 – 1.54537
From female headed household	0.91056	0.23223 – 3.57018
Mother had enough food in the last week	1.47226	0.84695 – 2.55925
Mother adolescent (aged 14-19)	1.12210	0.73393 – 1.71555
Mother with some formal education	1.10661	0.79054 – 1.54904
Father with some formal education	1.53526	1.19813 – 1.96726

Table S3.13 Initial GEE model for outcome of: Mother discusses immunisation with spouse & family

Variable	OR	95% CI
Home visits intervention	2.57256	0.79195 – 8.35664
Male sex of child	1.03262	0.92855 – 1.14836
From urban community	0.77919	0.68089 – 0.89168
From female headed household	0.43714	0.22617 – 0.84489
Mother had enough food in the last week	2.28782	1.50700 – 3.47321
Mother adolescent (aged 14-19)	1.04519	0.83705 – 1.30508
Mother with some formal education	1.35785	1.14430 – 1.61125
Father with some formal education	1.35256	1.08470 – 1.68657

Table S3.14 Initial GEE model for outcome of: Mother involved in decision about immunising the child

Variable	OR	95% CI
Home visits intervention	1.93712	0.95208 – 3.94127
Male sex of child	0.90123	0.79202 – 1.02548
From urban community	0.91594	0.67214 – 1.24818
From female headed household	3.68106	2.10519 – 6.43657
Mother had enough food in the last week	1.59313	0.77873 – 3.25923
Mother adolescent (aged 14-19)	1.19351	0.85212 – 1.67167
Mother with some formal education	1.25395	0.80111 – 1.96277
Father with some formal education	0.82456	0.56685 – 1.19944
