# Supplementary material for: Impact of universal home visits on child health in Bauchi State, Nigeria: a stepped wedge cluster randomised controlled trial
Source: BMC Health Serv Res. 2021 Oct 12;21:1085. doi: 10.1186/s12913-021-07000-3 (PMC8513291; doi:10.1186/s12913-021-07000-3)
Supplement: Supplementary file 4 — Additional file 4. Results of sensitivity analysis, excluding data from wards in wave 1 and wave 4. The file contains tables showing results from sensitivity analysis on the same outcome indictors included in the main manuscript but excluding data from wards in wave 1 and wave 4 [file 12913_2021_7000_MOESM4_ESM.pdf]

Results of sensitivity analysis, excluding data from wards in wave 1 and wave 4

Table A4.1: GEE modelling for intervention effect on prevalence of diarrhoea and on intermediate outcomes in children aged 12-18 months in intervention and control groups, including data from waves 2 and 3 only

Outcomes	Proportion (n/N)		Robust OR (95% CI)	
	Intervention	Control	Modelled with intervention alone[1]	Modelled with other characteristics[2]
Primary outcomes				
Diarrhoea in the last 15 days	0.149 (100/671)	0.328 (1187/3614)	**0.40 (0.26 – 0.62)**	**0.41 (0.28 – 0.61)**
Bloody diarrhoea in the last 15 days	0.022 (15/667)	0.063 (226/3579)	**0.25 (0.11 – 0.57)**	**0.26 (0.12 – 0.57)**
Intermediate outcomes				
Mother mentions lack of hygiene as a cause of diarrhoea	0.626 (465/743)	0.401 (1670/4169)	**2.77 (1.23 – 6.22)**	**2.83 (1.21 – 6.60)**
Household has better hygiene (no garbage, sewage or excreta observed)	0.596 (428/718)	0.434 (1749/4029)	**2.85 (1.42 – 5.72)**	2.09[3] (0.73 – 5.99)
Household with clean, covered and raised drinking water container observed	0.464 (333/718)	0.254 (1023/4029)	**2.77 (1.66 – 6.45)**	**2.74 (1.63 – 4.59)**
Household treats drinking water	0.301 (216/717)	0.181 (727/4020)	**2.33 (1.21 – 4.48)**	**2.33 (1.21 – 4.48)**

OR=Odds Ratio, 95% CI = 95% confidence interval; **Bold** font indicates a difference significant at the 5% level.
The lower denominators for the primary outcomes of diarrhoea and bloody diarrhoea in the last 15 days in both intervention and control groups are because some mothers could not specify when the child last had an episode of diarrhoea, and these children were excluded from the analysis.

[1] The GEE model for each outcome included only the intervention variable and ward as cluster

[2] The GEE initial saturated model for each outcome included the intervention variable and other variables potentially related to the outcome shown in Table 3 (in main text), as well as ward as cluster. The OR and 95% CI are from the final GEE model for each outcome.

[3] The OR and 95% CI are those from the initial GEE model including the intervention variable and other variables; the intervention variable was not in the final model

Table A4.2: GEE modelling for intervention effect on management of diarrhoea and on intermediate outcomes in children aged 12-18 months in intervention and control groups, including data from waves 2 and 3 only

Outcomes	Proportion(n/N)		Robust OR (95% CI)	
	Intervention	Control	Modelled with intervention alone[1]	Modelled with other characteristics[2]
Primary outcomes[3]				
Given more fluids and continued feeding during last episode of diarrhoea	0.210 (21/100)	0.099 (117/1179)	2.34 (0.98 – 5.62)	**2.37 (1.11 – 5.06)**
Not given any medicine to stop diarrhoea during last episode of diarrhoea	0.450 (45/100)	0.076 (90/1181)	**8.60 (3.03 – 24.45)**	**9.09 (4.35 – 19.01)**
Intermediate outcomes				
Mother thinks child with diarrhoea should be given more fluid and continued feeding	0.280 (208/743)	0.120 (500/4169)	**2.87 (1.52 – 5.44)**	**2.94 (1.26 – 6.89)**
Mother would not give a child medicines to stop diarrhoea	0.315 (234/743)	0.034 (140/4169)	**9.59 (4.59 – 20.04)**	**9.59 (4.59 – 20.04)**

OR=Odds Ratio, 95% CI = 95% confidence interval; **Bold** font indicates a difference significant at the 5% level.

[1] The GEE model for each outcome included only the intervention variable and ward as cluster

[2] The GEE initial saturated model for each outcome included the intervention variable and other variables potentially related to the outcome shown in Table 3 (in main text), as well as ward as cluster. The OR and 95% CI are from the final GEE model for each outcome.

[3] Among children with diarrhoea in the last 15 days

Table A4.3: GEE modelling for intervention effect on immunisation status and on intermediate outcomes in children aged 12-18 months in intervention and control groups, including data from waves 2 and 3 only

Outcomes	Proportion(n/N)		Robust OR (95% CI)	
	Intervention	Control	Modelled with intervention alone[1]	Modelled with other characteristics[2]
Primary outcome				
Fully immunised	0.637 (480/754)	0.489 (2037/4167)	2.22 (0.90 – 5.50)	2.21[3] (0.77 – 6.39)
Intermediate outcomes				
Mother thinks it is worthwhile to immunise children	0.968 (718/742)	0.969 (4006/4134)	1.47 (0.39 – 5.59)	1.97[3] (0.56 – 6.94)
Mother discusses immunisation with spouse and family	0.950 (706/743)	0.934 (3892/4169)	1.81 (0.90 – 3.63)	**2.41 (1.29 – 4.48)**
Mother involved in decision about immunising the child	0.229 (173/755)	0.147 (610/4195)	1.27 (0.53 – 3.05)	1.42[3] (0.41 – 4.97)

OR=Odds Ratio, 95% CI = 95% confidence interval

[1] The GEE model for each outcome included only the intervention variable and ward as cluster

[2] The GEE initial saturated model for each outcome included the intervention variable and other variables potentially related to the outcome shown in Table 3 (in min text), as well as ward as cluster. The OR and 95% CI are from the final GEE model for each outcome.

[3] The OR and 95% CI are those from the initial GEE model including the intervention variable and other variables; the intervention variable was not in the final model.
