# Supplementary material for: Impact of universal home visits on child health in Bauchi State, Nigeria: a stepped wedge cluster randomised controlled trial
Source: BMC Health Serv Res. 2021 Oct 12;21:1085. doi: 10.1186/s12913-021-07000-3 (PMC8513291; doi:10.1186/s12913-021-07000-3)
Supplement: Supplementary file 5 — Additional file 5. Other initiatives in Toro LGA potentially related to the measured child health outcomes. This file contains a description and relevant web links to view details about other initiatives in Toro LGA during the period of the home visits [file 12913_2021_7000_MOESM5_ESM.pdf]

Additional file 5: Other initiatives in Toro LGA potentially related to the measured child health outcomes

During the period of our project there were several other donor-supported initiatives implemented in Toro LGA.

(a) The USAID-funded Maternal and Child Survival Program (MCSP) (https://pdf.usaid.gov/pdf_docs/PA00TMN1.pdf). This focussed specifically on improving routine immunisation coverage. The program mostly aimed to improve facility- based services and it covered all wards in the LGA. It seems unlikely that this project had any confounding effect. Also, according to our findings, our home visits intervention did not significantly improve routine child immunisation.

(b) The BORN project implemented by Plan international with support from Global Affairs Canada. The project mainly focussed on maternal and newborn child health. The project worked in all the wards in Toro and in all other LGAs of the state and would not have affected differently the intervention and control groups in our wards.

(c) The Nigerian State Health Investment Programme (NSHIP) (https://dc.sourceafrica.net/documents/120566-NSHIP-PROJECT-INFORMATION-DOCUMENT-PID.html), a project of the Federal Government of Nigeria, supported through credits from the World Bank. The programme was implemented across Bauchi State, including Toro LGA. It focussed on improving infrastructure, human resources and supplies at primary health care facilities; it did not include any community-based preventive or promotive activity. It is unlikely to have influenced household actions for childhood diarrhoea prevention or management, and it was not implemented differentially in the wards implementing the home visits intervention.

(d) Throughout the period of our project, the Bauchi State Primary Health Care Development Agency continued to receive support from WHO and UNICEF for routine PHC activities. These took place throughout the State, including in all wards of Toro LGA throughout the period of the home visits implementation, and would not have differentially influenced outcomes in intervention and control groups.

(e) Breakthrough ACTION Nigeria (https://breakthroughactionandresearch.org/tag/nigeria/) is a USAID-funded project in some areas of Bauchi State. It aims to increase priority health behaviours in several domains: malaria; maternal, newborn, and child health and nutrition; family planning and reproductive health; and tuberculosis. This project was at the planning stage with no field implementation during the period of implementation of our home visits.
